# A potential osteogenic role for microRNA-181a-5p during palatogenesis

**DOI:** 10.1093/ejo/cjad037

**Published:** 2023-07-16

**Authors:** Christian Schoen, Marjon Bloemen, Carine E L Carels, Gerald W Verhaegh, Rene Van Rheden, Laury A Roa, Jeffrey C Glennon, Johannes W Von den Hoff

**Affiliations:** Department of Orthodontics and Craniofacial Biology, Radboud Institute for Molecular Life Sciences, Radboud University Medical Center, Nijmegen, the Netherlands; Department of Orthodontics and Craniofacial Biology, Radboud Institute for Molecular Life Sciences, Radboud University Medical Center, Nijmegen, the Netherlands; Department of Human Genetics and Department of Oral Health Sciences, KU Leuven and orthodontic clinic, University Hospitals KU Leuven, Belgium; Department of Urology, Radboud Institute for Molecular Life Sciences, Radboud University Medical Center, Nijmegen, the Netherlands; Department of Orthodontics and Craniofacial Biology, Radboud Institute for Molecular Life Sciences, Radboud University Medical Center, Nijmegen, the Netherlands; Department of Orthodontics and Craniofacial Biology, Radboud Institute for Molecular Life Sciences, Radboud University Medical Center, Nijmegen, the Netherlands; MERLN Institute for Technology—Inspired Regenerative Medicine, Maastricht University, the Netherlands; Conway Institute of Biomolecular and Biomedical Research, School of Medicine, University College Dublin, Ireland; Department of Cognitive Neuroscience, Donders Institute for Brain, Cognition and Behaviour, Radboud University Medical Center, Nijmegen, the Netherlands; Department of Orthodontics and Craniofacial Biology, Radboud Institute for Molecular Life Sciences, Radboud University Medical Center, Nijmegen, the Netherlands

## Abstract

**Background:**

In a previous study, we found that the highly conserved hsa-miR-181a-5p is downregulated in palatal fibroblasts of non-syndromic cleft palate-only infants.

**Objectives:**

To analyze the spatiotemporal expression pattern of mmu-miR-181a-5p during palatogenesis and identify possible mRNA targets and their involved molecular pathways.

**Material and methods:**

The expression of mmu-miR-181a-5p was analyzed in the developing palates of mouse embryos from E11 to E18 using qPCR and ISH. Mouse embryonic palatal mesenchyme cells from E13 were used to analyze mmu-miR-181a-5p expression during osteogenic differentiation. Differential mRNA expression and target identification were analyzed using whole transcriptome RNA sequencing after transfection with a mmu-miR-181a-5p mimic. Differentially expressed genes were linked with underlying pathways using gene set enrichment analysis.

**Results:**

The expression of mmm-miR-181a-5p in the palatal shelves increased from E15 and overlapped with palatal osteogenesis. During early osteogenic differentiation, mmu-miR-181a-5p was upregulated. Transient overexpression resulted in 49 upregulated mRNAs and 108 downregulated mRNAs (adjusted *P*-value < 0.05 and fold change > ± 1.2). Ossification (*Stc1*, *Mmp13*) and cell-cycle-related GO terms were significantly enriched for upregulated mRNAs. Analysis of possible mRNA targets indicated significant enrichment of Hippo signaling (*Ywhag*, *Amot*, *Frmd6* and *Serpine1*) and GO terms related to cell migration and angiogenesis.

**Limitations:**

Transient overexpression of mmu-miR-181a-5p in mouse embryonic palatal mesenchyme cells limited its analysis to early osteogenesis.

**Conclusion:**

Mmu-miR-181-5p expression is increased in the developing palatal shelves in areas of bone formation and targets regulators of the Hippo signaling pathway.

## Introduction

Non-syndromic cleft palate only (nsCPO) is one of the most common craniofacial birth defects with a significant associated healthcare burden (1). The risk of nsCPO is determined by genomic and environmental factors that disturb the complex outgrowth, elevation, fusion and differentiation of the secondary palate (2).

MicroRNAs (miRNAs) are small non-coding RNAs, which function as post-transcriptional repressors of gene expression, able to regulate multiple mRNA transcripts simultaneously (3). A wide range of biological processes is regulated by miRNAs, including epithelial-to-mesenchymal transition, migration, apoptosis, proliferation and differentiation (4). Hence, they are also essential during palatogenesis with dysregulation in mice resulting in cleft palate (5).

In a previous study, we found that homo sapiens (hsa-) miR-181a-5p is downregulated in palatal fibroblasts of nsCPO infants (6). The conserved miR-181 family has four members; miR-181a, miR-181b, miR-181c and miR-181d (7). Hsa-miR-181a/181b are intergenic, clustered and have two paralog copies (-1/-2) on chromosomes 1 and 9, while hsa-miR-181c/181d are clustered on chromosome 19. As one miRNA can target multiple mRNA transcripts, the biological functions of the miR-181 family members are diverse (8). During early craniofacial development, miR-181a is required for the survival of cranial neural crest cells in the pharyngeal arches by regulating mitogen-activated protein kinase (MAPK) signaling (9). In later craniofacial development, miR-181a expression is linked to intramembranous ossification of mouse calvaria and retinal axon specification through transforming growth factor beta (TGF-β) and MAPK signaling (10,11).

To our knowledge, no specific study has been carried out on the role of miR-181a-5p in palatogenesis. Because miR-181a-5p is downregulated in palatal fibroblasts of nsCPO infants further research is warranted. Therefore, the purpose of this study was to investigate the function of mus musculus (mmu-) miR-181a-5p in palatogenesis by analyzing its spatiotemporal expression pattern in mouse embryos. Expression of mmu-miR-181a-5p was significantly upregulated in areas of palatal bone formation. Based on this, we further investigated its expression during early osteogenic differentiation of mouse embryonic palatal mesenchyme (MEPM) cells. We also analyzed its targets and associated molecular pathways after overexpression with a miR-181a-5p mimic.

## Material and methods

### Mice and tissue collection

Wild-type CD1 mice (Charles River, Wilmington, MA) were mated for 4 h, and the presence of a vaginal plug was designated as embryonic day (E) 0. Five pregnant mice were sacrificed at each of the embryonic stages E11–E18. Palatal shelf dissection of four to five embryos from four different mothers was performed under the microscope, pooled per litter and used for expression analysis of miRNAs and mRNAs. Additional embryo heads were fixed in 4% paraformaldehyde overnight at 4°C for histological investigation. MEPM cells were isolated from E13 palatal shelves by enzymatic digestion, plastic adherence and mesenchyme-specific medium and shown to have osteogenic potential (12). The cells from embryos of three different mothers were mixed and frozen in liquid nitrogen after two passages. The animal study complied with the ARRIVE guidelines and was approved by the board for animal experiments of Radboud University, Nijmegen (RUDEC 2015-0080).

### Cell culture and mimic transfection

MEPM cells were seeded (100,000 cells/well) in 12-well plates in proliferation medium composed of minimum essential medium α (MEMα, Gibco) with 10% fetal bovine serum (FBS, Gibco), 100 U/ml penicillin, 100 μg/ml streptomycin (1% P/S, Sigma) and cultured at 37°C in a humidified atmosphere of 5% CO_2_ in air. Upon 90% confluency, the culture medium was switched to osteogenic differentiation medium composed of minimum essential medium α (MEMα, Gibco), 10% FBS, 1% P/S, 10 mM β-glycerophosphate, 10 nM dexamethasone and 50 μg/ml vitamin C. A standard mimic for mmu-miR-181a-5p and negative control/scramble (Ambion) were ordered and used for transfection (10 nM/well) using Lipofectamine RNAiMAX reagent (Invitrogen) and Opti-MEM serum-free medium (Gibco) according to the manufacturer’s protocol. Cells were harvested after 24 h, 48 h and 7 days. Cultures for the analysis of RT-qPCR were grown in triplicate and for RNA sequencing in duplicate.

### Histological analysis

Embryo heads were embedded in paraffin, and sectioned (5 µm). Frontal sections from the heads were stained with hematoxylin–eosin for a general tissue survey. For *in situ* hybridization (ISH), a double digoxygenin (DIG)-labeled mmu-miR-181a-5p probe (Dig-AcuCacCgaCagCguTgaAuguT-DIG, capitals indicating locked nucleic acid modifications and lower case indicating 2’OME RNA from Exiqon was used according to van Scheppingen *et al* (13). A scramble probe was used as negative control (Exiqon). Deparaffination was followed by proteinase-K treatment. Hybridization was detected with alkaline phosphatase (AP) labeled anti-DIG (Roche Applied Science). NBT (nitro-blue tetrazolium chloride)/BCIP (5-bromo-4-chloro-3ʹ-indolyphosphate *p*-toluidine salt) was used as a chromogenic substrate for AP. See Supplementary information for details. For alkaline phosphatase (Alp) staining, paraffin sections were deparaffinated and rehydrated. After incubation in 0.1 M Tris/HCL buffer (pH 9.5) containing 50 mM MgCL2 and 100 mM NaCl for 10 min, sections were transferred to the same buffer containing NBT/BCIP (Roche Applied Science) at 37°C for 20 min.

### RNA isolation and qPCR

Total RNA from MEPM cells and dissected palates were isolated using the miRNeasy Mini Kit (Qiagen) according to the manufacturer’s protocol and quantified by the NanoDrop ND-2000 (Thermo Scientific). cDNA was generated from 500 ng small RNA using the miScript II RT Kit (Qiagen) according to the manufacturer’s protocol. Reverse transcription-quantitative polymerase chain reaction (PCR) was performed in duplicate in a total reaction volume of 10 μl containing 5 μl SYBR Green PCR Master Mix (Qiagen), 1 μl of cDNA, 2 μl of RNAse-free water and 2 μl of the primer mix. For miRNAs, 1 ul of 5 uM forward primer and 1 ul of 10× miScript Universal Primer (Qiagen) were used as the primer mix. cDNA amplification was performed in the Rotor-Gene Q (Qiagen) with an initial activation step at 95°C for 15 min followed by 40 cycles of 94°C for 15 s, 55°C (60°C for mRNAs) for 30 s and 70°C for 30 s. Primers were obtained from Biolegio and their sequences are shown in Supplementary Table 1. Expression was calculated from the mean Ct values of the duplicates from each sample using the delta Ct method (2^−ΔCt^). *U6 snRNA* and *GAPDH* were used as the internal control for miRNA and mRNA expression, respectively.

### RNA sequencing

Total RNA was isolated from MEPM cells 24 h after mimic (*n* = 2) or scramble transfection (*n* = 2). RNA sequencing libraries were prepared using the KAPA RNA HyperPrep Kit with RiboErase (HMR) (KAPA Biosystems) according to protocol. Fragmentation and priming were performed at 94°C for 6.5 min. First-strand synthesis, second-strand synthesis and A-tailing were performed according to protocol. For the adapter ligation, a 7 μM stock was used (NextFlex DNA barcodes, Bioo Scientific). First, and second post-ligation cleanup was performed according to protocol. For the library amplification, six cycles were used. The library amplification cleanup was performed using a 0.8× bead-based cleanup. Library size was determined using the High Sensitivity DNA bioanalyzer (Agilent Technologies), library concentration was measured using the DeNovix dsDNA High Sensitivity Assay (DeNovix). Paired-end sequencing was performed using an Illumina NextSeq 500. Low-quality ﬁltering and adapter trimming were performed using Trim Galore! v0.4.5 (Babraham Bioinformatics), a wrapper tool around the tools Cutadapt v1.18 and FastQC v0.11.8 (Babraham Bioinformatics). Reads were mapped to a human reference genome (GRCh38.95, Ensembl) with Star v2.7.5a, resulting in BAM files. BAM files were counted with HTSeq [HTSeq-count tool v0.11.0] with default parameters using a complementary gtf file, containing annotation for GRCh38.95 (Ensembl). Counts were normalized using gene length and transcripts per million were produced. MultiQC (quality control) was used to combine results and quality checks of all the samples.

### Bioinformatic analysis

Differential gene expression analysis was carried out with DESeq2 v1.22.0 in R v3.5.3, with internal statistical and normalization method (correction for multiple testing with Benjamini–Hochberg) using a cutoff value of at least 5 counts per sample per gene. Genes with adjusted *P* < 0.05 and minimum fold change of ±1.2 between the negative control and mimic samples were considered to be differentially expressed. Kegg pathway/Gene Ontology enrichment analysis was performed using ShinyGO v0.75 (adjusted *P* < 0.05) (14). Three miRNA target prediction tools were used: sequence-based TargetScan 7.2, energy-based microT-CDS 5.0 and machine learning-based MirDB (15–17). Threshold values for positive prediction were an aggregate probability of conserved targeting (Pct) score > 0.36 for TargetScan, a miRNA target prediction (miTG) score > 0.7 for microT-CDS and a prediction score > 50 for MirDB. Additional experimentally validated mmu-miR-181a-5p target interactions from miRTarBase 8.0 were also included.

### Statistical analysis

Data are presented as mean ± standard deviation. Normality was tested with the D’Agostino–Pearson normality test. Differences between the groups were evaluated by the paired Student’s t-test and the analysis of variance. *Post hoc* comparisons were made using the Bonferroni test. Differences were considered significant if *P* < 0.05. All statistical tests were performed with Graphpad Prism software (Graphpad Software, La Jolla, CA, USA).

## Results

### Mmu-miR-181a-5p expression increases during palatogenesis

**Figure 1:**
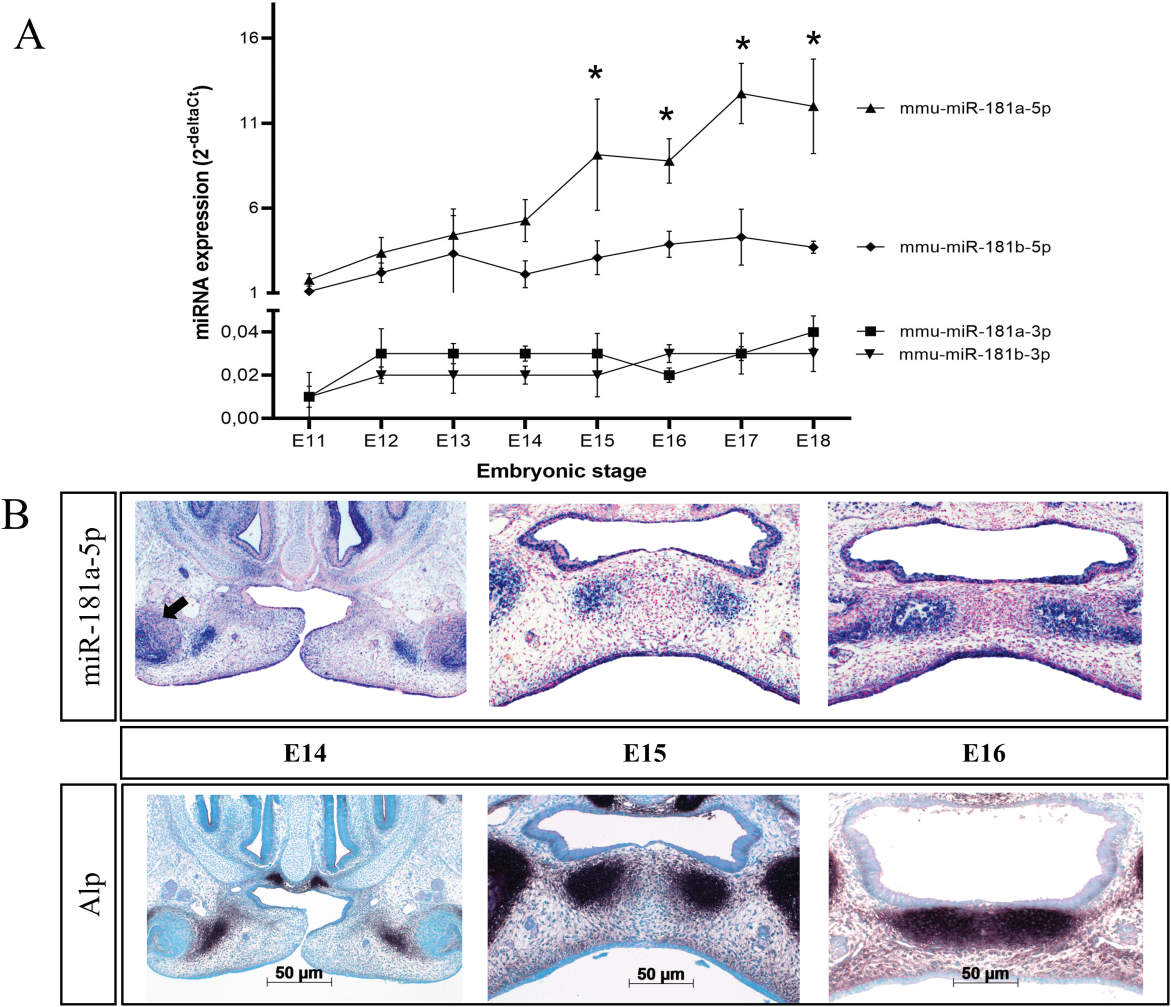
(A) Expression of the mature strands of the mmu-miR-181a/b-1/2 cluster on chromosomes 1 and 2 from E11 to E18. To show the relative amount of each miRNA, delta Ct expression values are shown. Each miRNA was normalized to U6. Data are represented as the mean ± SD (*n* = 4). *P < 0.05 comparing the mmu-miR-181a-5p over time. (B) Alp and mmu-miR-181a-5p staining, using a chromogenic substrate and ISH, respectively. Scale bar represents 50 um. Arrow = beginning expression in developing molar germ. Images are representative of three independent repeats and contrast was optimized between samples.

To analyze the temporal expression pattern of mmu-miR-181a-5p, we investigated the expression by qPCR in dissected palatal shelves from E11 to E18 (Figure 1A). As miR-181a-5p is transcribed as part of a polycistronic miRNA gene, all members were analyzed. Expression of mmu-miR-181a/b-3p remained stable and was about 114 times lower than the 5p strand, suggesting the latter as the predominant strand within the developing palate. Mmu-miR-181a/b-5p were expressed at similar levels until E15, when mmu-miR-181a-5p started to be upregulated. Mmu-miR-181a-5p expression increased by 6.8-fold from E11 to E18. Expression of mmu-miR-181a-5p was significantly upregulated from E15 onwards compared to E11.

### Mmu-miR-181a-5p is expressed in areas of palatal osteogenesis

To analyze the spatial expression pattern of mmu-miR-181a-5p, we performed ISH. The miRNA was constitutively expressed in the oral and nasal epithelium from E11 to E18. Sections from E14, E15 and E16 are shown in Figure 1B. No difference was found in the staining of the epithelium during fusion or disappearance of the midline epithelial seam. From E14 on, additional mmu-miR-181a-5p expression overlapped with Alp activity in the lateral osteogenic areas of the palatal shelves and the tooth germs (Figure 1B). After fusion, at E15, two separated more medial areas of joint mmu-miR-181a-5p and Alp activity were detected, and the lateral staining had increased. At E16, the medial and lateral areas of mmu-miR-181a-5p expression started to connect, while both medial sides remain separated in contrast to Alp expression.

### Mmu-miR-181a-5p is upregulated during osteogenic differentiation of MEPM cells

**Figure 2:**
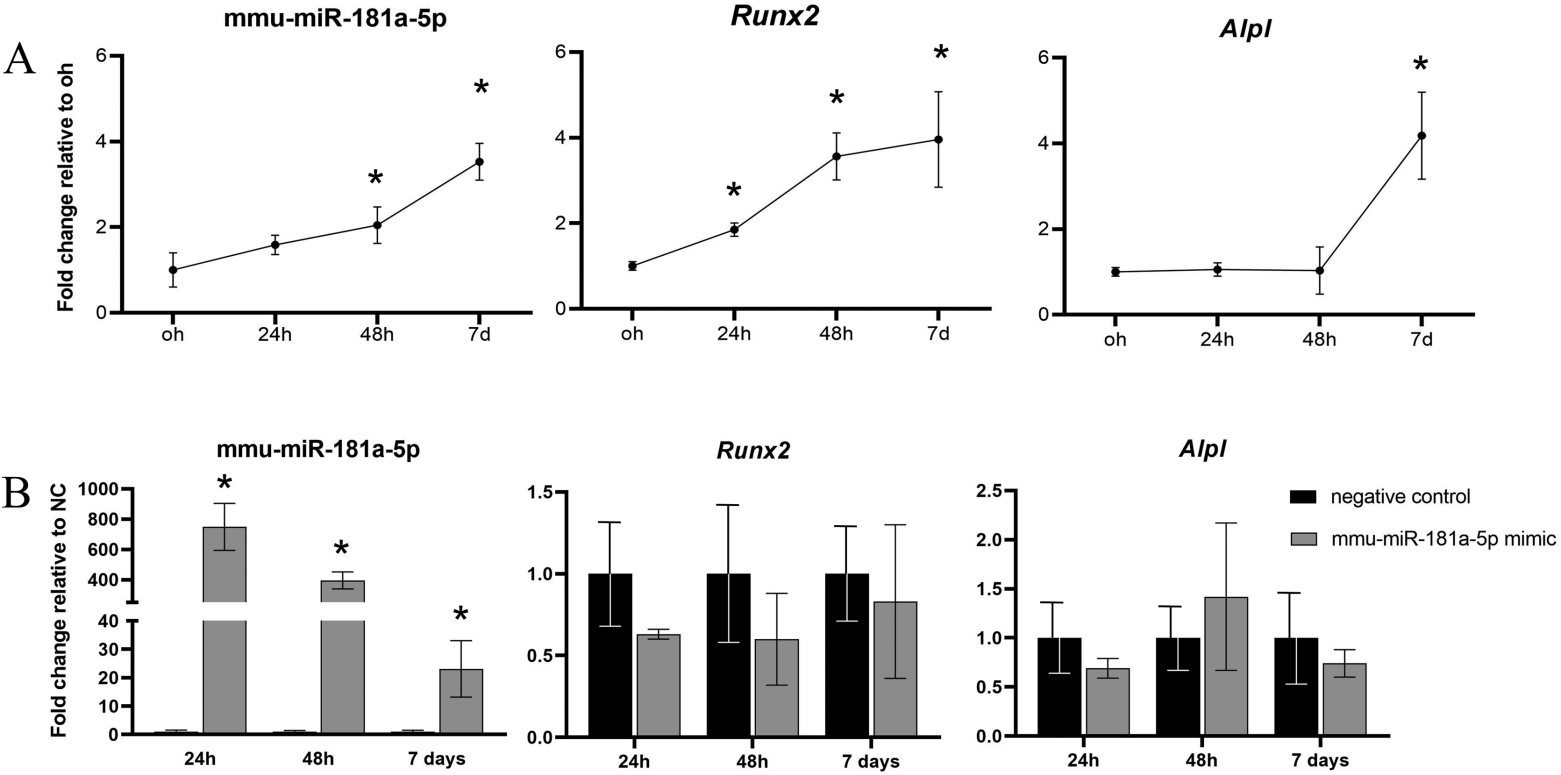
(A) Expression of Runx2, Alp and mmu-miR-181a-5p from 0 h to day 7. To show the increase in expression after osteogenic stimulation the fold change relative to 0 h was shown. The miRNA was normalized to U6 and the mRNAs to Gapdh. Data are represented as the mean ± SD (*n* = 3). * *P* < 0.05 comparing the fold change to 0 h. (B) Expression 24 h after transfection of mimic and negative control. To show the effect of the mmu-miR-181a-5p mimic, fold change of each miRNA or mRNA was shown relative to the negative control. The miRNA was normalized to U6 and the mRNAs to *Gapdh*. Data are represented as the mean ± SD (*n* = 3). * *P* < 0.05.

As mmu-miR-181a-5p was only significantly upregulated from E15, which coincides with its increased expression in osteogenic regions, mmu-miR-181a-5p expression was also investigated during MEPM cell osteogenic differentiation (Figure 2A). During MEPM cell osteogenic differentiation, endogenous expression levels of mmu-miR-181a-5p gradually increased over time compared to day 0 cultures, reaching significance at 48 h. Runt-related transcription factor 2 *(Runx2)*, a marker of early osteogenic differentiation, was significantly increased from 24 h on. *Alpl*, a marker of bone matrix production, remained low up to 48 h and then increased significantly at day 7. Bone gamma-carboxyglutamate protein *(Bglap)*, a marker of bone matrix mineralization, was nearly undetectable (Ct value > 38) until day 7 when its expression was still very low (Ct = 35–36). The data thus indicate that mmu-miR-181a-5p is also upregulated during the osteogenic differentiation of MEPM cells.

### Transient mmu-miR-181a-5p overexpression in MEPM cells

**Figure 3:**
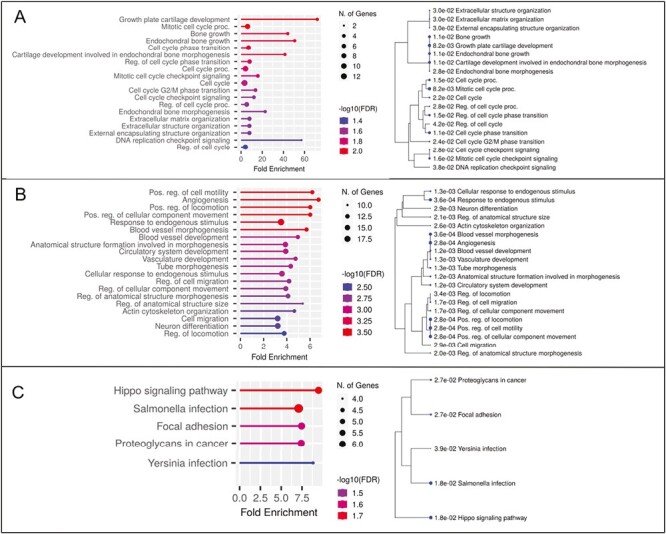
Gene Ontology enrichment analysis of differentially expressed genes. Left column: Significantly enriched terms sorted based on *P*-value with visualization of the fold enrichment on the x-axis. Bigger dots indicate a larger number of genes. Right column: hierarchical clustering tree based on shared genes. Bigger dots indicate more significant *P*-values. (A) upregulated genes GO biological processes, (B) downregulated genes GO biological processes (top 20), (C) downregulated genes KEGG pathways.

**Figure 4:**
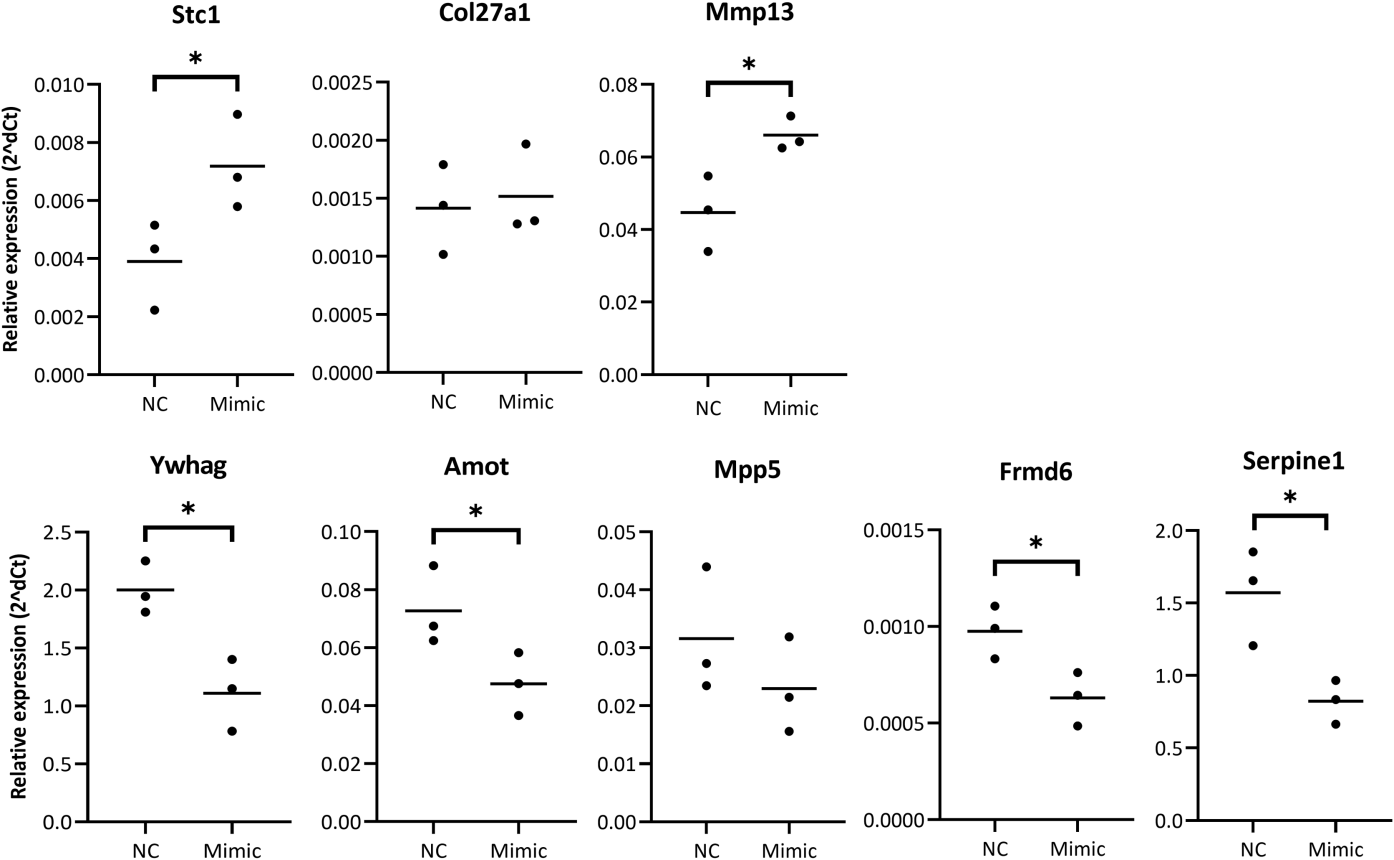
Expression of targets 24 h after transfection of mimic and negative control. The mRNAs were normalized to *Gapdh*. Data are represented as a dot plot (*n* = 3). * *P* < 0.05

**Figure 5:**
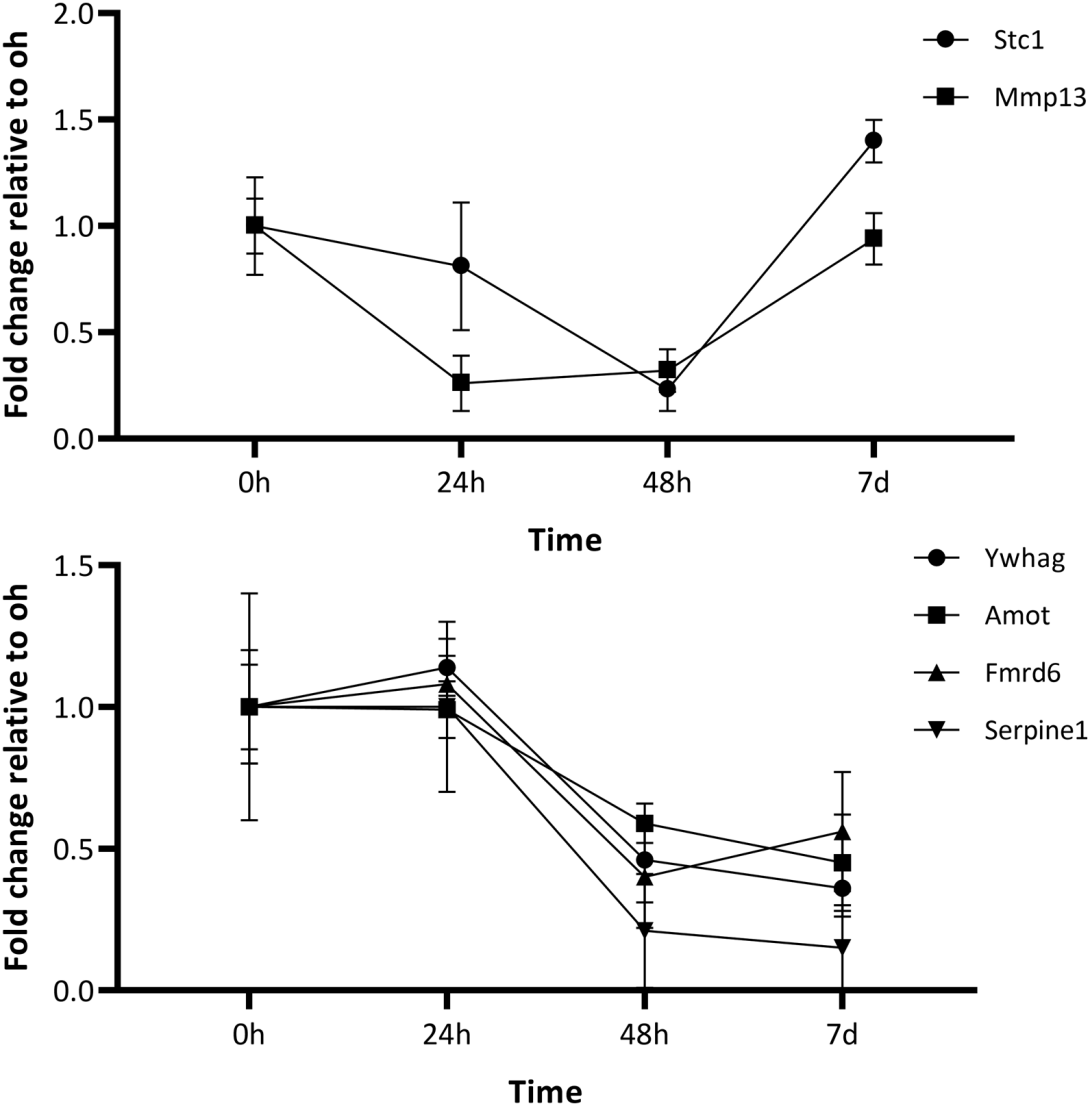
Expression of significantly upregulated and downregulated mRNAs from 0 h to day 7. To show the increase in expression after osteogenic stimulation the fold change relative to 0 h was shown. The mRNAs were normalized to *Gapdh*. Data are represented as the mean ± SD (*n* = 3).

Following transfection of MEPM cells with a mmu-miR-181a-5p mimic, we found a mean 749-fold increase in miRNA levels at 24 h compared to endogenous levels in non-transfected cells (Figure 2B). Overexpression of mmu-miR-181a-5p persisted until day 7, although expression levels decreased rapidly. No significant changes were detected in *Runx2* or *Alpl* expression (Figure 2B). As no significant changes were detected in *Runx2* or *Alpl* expression, we performed RNA sequencing after 24 h of transfection to detect differences at the whole transcriptome level. In total, 49 mRNAs were upregulated and 108 mRNAs were downregulated (fold change > ±1.2, adjusted *P*-value < 0.05, see Supplementary information). Overall, only small changes in gene expression were detected (Supplementary Figure 1). Upregulated mRNAs showed significant enrichment for 19 GO biological process terms and none for KEGG pathways (Figure 3A). Hierarchical clustering of the GO terms showed that the related biological terms are mainly cell cycle-related and ossification-related processes. The ossification group contained three upregulated genes, *Stc1*, *Mmp13* and *Col27a1*. Only *Stc1* and *Mmp13* upregulation were validated with qPCR analysis (Figure 4). To limit the analysis of non-specifically downregulated genes we included only those mRNAs that are predicted targets of mmu-miR-181a-5p (from at least one out of three prediction software or with experimental evidence). A total of 76 downregulated genes were included, of which *Tgfbi* and *Rgs4* have been previously identified as a target of mmu-miR-181a-5p during osteogenic differentiation (10). The downregulated mRNAs were then subjected to GO and KEGG pathway analyses. There was significant enrichment of 107 GO biological terms and four KEGG pathways (Figure 3B and C). Hierarchical clustering of the top 20 enriched GO terms divided the biological processes into predominantly cell migration-related and angiogenesis-related processes. Downregulated genes belonging to the Hippo signaling pathway were selected for qPCR validation (Figure 4). Except for *Mpp5*, the results were consistent with the RNA-seq results (Figure 4). Further qPCRs were performed to analyze whether these findings correlate with miR-181a-5p expression during MEPM osteogenic differentiation (Figure 5). All validated targets were significantly downregulated at 48 h, the time point at which miR-181a-5p expression is upregulated. For *Stc1* and *Mmp13*, expression decreased up to 48 h after which it increased.

## Discussion

Hsa-miR-181a-5p is downregulated in palatal fibroblasts of infants with cleft palate only (6). As miR-181a-5p is highly conserved between humans and mice (18), we analyzed its expression by qPCR in mouse palatal shelves from E11 to E18. The expression was upregulated from E16 onwards. The only other miR-181a-5p expression study in palatal shelves was based on microarray data from E12 to E14 and showed that it was downregulated in mice from E13 to E14 (19). We did not find a significant difference between these two time points. This difference may be due to the fact that microarray data, in contrast to qPCR data, are less robust and reliable (20). Also, in our ISH staining of E13 and E14 (data not shown), we did not find qualitative differences. Mmu-miR-181a-5p expression in the epithelium is constant in our study and it has previously been identified to regulate keratinocyte homeostasis (21). From E15 onwards we did find new areas of miR-181a-5p expression within the palatal shelves overlapping with bone-forming areas and also in the developing tooth germs. As the developing tooth germs fall outside our dissected palatal shelves, the new expression coinciding with bone-forming areas corroborates the upregulated expression in the qPCR data. In calvarial tissues, mmu-miR-181a was also increasingly expressed during intra-membranous ossification from day 0 to day 42 (10). Furthermore, we showed that mmu-miR-181a-5p is upregulated during the osteogenic differentiation of MEPM cells. Our data thus suggest that mmu-miR-181a is upregulated during palatal bone formation.

While the 5p strand of miR-181a was upregulated, we found that both mmu-miR-181a/b-3p strands were expressed at very low levels in the developing palatal shelves and thus probably have a limited biological function. Since hsa-miR-181a-3p inhibits osteogenesis in human bone marrow-derived mesenchymal stem cells (22), low expression levels may allow osteogenesis in the developing palate. In human posterior longitudinal ligament cells, the 5p strand and not the 3p strand was also involved in the regulation of osteogenesis (23). This suggests that miR-181a-5p is the predominant strand of the cluster as palatogenesis progresses.

To detect the effect of mmu-miR-181a-5p upregulation during MEPM cell osteogenic differentiation we used whole transcriptome RNA sequencing. The small effects of mmu-miR-181a-5p overexpression are likely due to the fact that miRNAs generally only modestly alter the expression of target genes. However, the cumulative effect of targeting many genes simultaneously can lead to significant alterations of biological processes. This is in agreement with Zheng et al., who only detected modest changes in mRNA expression after miR-181 overexpression (24). We performed separate enrichment analysis for up- and downregulated genes as this is a more powerful approach for identifying pertinent pathways (25).

Downregulated genes were cross-referenced with experimental evidence or target prediction software before enrichment analysis. This is because non-specific changes may occur due to the saturation of miRISC complexes and displacement of other endogenous miRNAs leading to some downregulated genes appearing functionally important (26). By analyzing the cross-referenced downregulated genes only 24 h after transfection, these genes likely represent direct targets of mmu-miR-181a-5p during osteogenic differentiation of MEPM cells. This is shown by the high number of downregulated genes to be experimentally validated or predicted targets. Similar to Bhushan *et al*., we identified that mmu-miR-181a-5p targets the same mRNAs, *Tgfbi* and *Rgs4*, in MEPM cells and MC3T3 cells during osteogenic differentiation (10). Unlike in posterior longitudinal ligament cells and DDCs we did not identify any difference in *Pbx1* and *Pten* mRNA levels (23,24). Interestingly, Hippo signaling was identified as the most significantly enriched pathway. The genes involved were: *Ywhag, Amot, Fmrd6, Mpp5* and *serpine1* and all are downregulated when miR-181a5p is upregulated during osteogenic differentiation of MEPM cells. All but the former are active during Hippo signaling and function to limit the nuclear localization of *Yap/Taz* and thus inhibiting their activity.

*Yap/Taz* stimulates mineralization of the bones of the secondary palate with a similar expression pattern as mmu-miR-181a-5p (27). Furthermore, *Yap/Taz* promotes osteogenesis of cranial neural crest-derived cells (28). Deletion of *Yap/Taz* results in undersized bones of the secondary palate due to decreased expression of the downstream targets *Lbsp* and *Phex*. Both are involved in mineralization. We also identified upregulated expression of *Phex* in our RNA sequencing data but this was not significant (data not shown). The two upregulated genes *Mmp13* and *Stc1* are also linked to the initiation and stimulation of mineralization during intramembranous ossification (29–31). In our study, *Mmp13* and *Stc1* expression decrease up to 48 hours after which they increase up to day 7. The delay between mmu-miR-181a-5p increased expression and that of both osteogenesis-related genes may be due to the gradual increase of expression of the miRNA. Also, *Mmp13* is downstream of the downregulated targets. A recent study reported that increased nuclear *Yap1*, through inhibition of the Hippo signaling pathway, led to increased levels of *Mmp13* (32). *Mmp13* has also been identified as a downstream target of TGF-β during palatogenesis, while its absence can result in a cleft of the secondary palate. However, expression of *Mmp13* was only detected in the mesenchyme and epithelium around the midline epithelial seam not coinciding with miR-181a-5p expression (33). *Stc1* is downstream to many pathways but to date the association between the gene and the Hippo signaling pathway has not yet been analyzed (34).

Disruption of secondary palate ossification might result in the submucous cleft palate as in *Tbx22* and *Bmpr1a* deletion (35). Disturbed or absent palatal osteogenesis can also lead to complete cleft palate such as in *Hoxa2*, *Osr2* and *Meis2* deletion (35–38). Loss of function mutations in *YAP1* has been identified in families with CLP (39). It may therefore be possible that disturbed miR-181a-5p expression influences the risk cleft palate.

## Conclusion

In this study, we provide evidence that mmu-miR-181a-5p expression is concurrent with palatal osteogenesis and early osteogenic differentiation of E13 MEMP cells. Transient overexpression of a mmu-miR-181a-5p mimics upregulated *Mmp13* and *Stc1*, which are both associated with bone mineralization. We validated previous findings that mmu-miR-181a-5p targets *Tgfbi* during osteogenic differentiation and identified Hippo signaling as a possible pathway through which it may regulate osteogenic differentiation in the developing palate.

## Supplementary Material

cjad037_suppl_Supplementary_Material

## Data Availability

The data of the statistically significant up and downregulated mRNAs from the RNA-seq experiment can be found in the supplementary information.
