# Supplementary material for: A potential osteogenic role for microRNA-181a-5p during palatogenesis
Source: Eur J Orthod. 2023 Jul 16;45(5):575–83. doi: 10.1093/ejo/cjad037 (PMC10756689; doi:10.1093/ejo/cjad037)
Supplement: cjad037_suppl_Supplementary_Material [file cjad037_suppl_supplementary_material.docx]

**Protocol for ISH**

Sections were deparaffinized using xylene 3x 3min, ethanol 100% 2x 1min and ethanol 70% 1min and then washed with sterile water (Baxter, plastic bottles) 3x. Sections were then treated with proteinase K (0.5mg/ml) in buffer solution (50mM Tris, 1mM EDTA, 5 mM CaCl_2_, 0.5% triton X100, pH=8) for 10 min. After proteinase K treatment sections were incubated in hybridization mix (50% (vol/vol) deionized formamide, 600 mM NaCl, 10 mM HEPES buffer, pH 7.5, 1 mM EDTA, 5x Denhardt’s reagent and 200 ug/ml denatured herring sperm DNA (Sigma)) for 15min at 70°C. The probes (100nM) were heated in the hybridization mix for 5 min at 95^o^C after which they were pipetted on the sections and incubated for 1 hr at 70°C. Tissue sections were then washed at hybridization temperature with 2X SSC for 2min, 0.5X SSC for 2min and 0.2X SSC for 1min. Further washing was done with PBS (sterile flask) at room temperature 2x. Slides were then blocked for 15min with PBS containing 1% (w/v) BSA, 0.02% Tween 20 and 1:100 normal serum. Slides were incubated with sheep anti-DIG alkaline phosphatase (Roche) in the blocking buffer for 1hr at room temperature and then washed in PBS 3x and TBS-0.1% Tween 2x. Color was developed with NBT/BCIP (1:50 in NTM-T buffer (100 mM Tris, pH 9.5; 100 mM NaCl; 50 mM MgCl2; 0.05% Tween20). After colour reacton slides were washed with demi water, dried and mounted in vectamount.

**Supplementary table 1.**

Primer sequences used for RT-qPCR

| **Gene Name** | **Forward primer (5’-3’)** | **Reverse Primer (3’-5’)** |
| --- | --- | --- |
| *Mir-181a-5p* | AACATTCAACGCTGTCGGTGAGT |  |
| *MiR-181a-3p* | ACCATCGACCGTTGATTGTACC |  |
| *MiR-181b-5p* | AACATTCATTGCTGTCGGTGGGTT |  |
| *MiR-181b-3p* | CTCACTGAACAATGAATGCAA |  |
| *U6* | CGCTTCGGCAGCACATATAC | AAAATATGGAACGCTTCACGA |
| *Gapdh* | GGCAAATTCAACGGCACA | GTTAGTGGGGTCTCGCTCCTG |
| *Alpl* | GCACCTGCCTTACCAACTCT | GTGGAGACGCCCATACCATC |
| *Runx2* | CGGACGAGGCAAGAGTTTCA | GGATGAGGAATGCGCCCTAA |
| *Bglap* | CCGGGAGCAGTGTGAGCTTA | CCATACTGGTCTGATAGCTC |
| *Stc1* | ACGAGGCGGAACAAAATGATT | TGCACTTTAAGCTCTCTTTGACA |
| *Col27a1* | GCACCTTCCAAAGCCTCTAGT | CTGAAGCGTTTTAGGGACACT |
| *Mmp13* | CTTCTTCTTGTTGAGCTGGACTC | CTGTGGAGGTCACTGTAGACT |
| *Ywhag* | GTGACCGAGCTGAACGAAC | GATGCTGCTGATGACCCTCC |
| *Amot* | CCGCCAGAATACCCTTTCAAG | CTCATCAGTTGCCCCTCTGT |
| *Mpp5* | TTTGGGCACCAGAATGATGC | AACAATTCCTTCTTCCGTGTCAA |
| *Frmd6* | CAAAGCCATGCAGGACCGT | GATGCCCCAAGTGACTTCGT |

**Supplementary figure 1**

Volcano plot. Red dots indicate significantly differentially expressed mRNAs (adjusted p-value <0,05 after correction of multiple testing

**
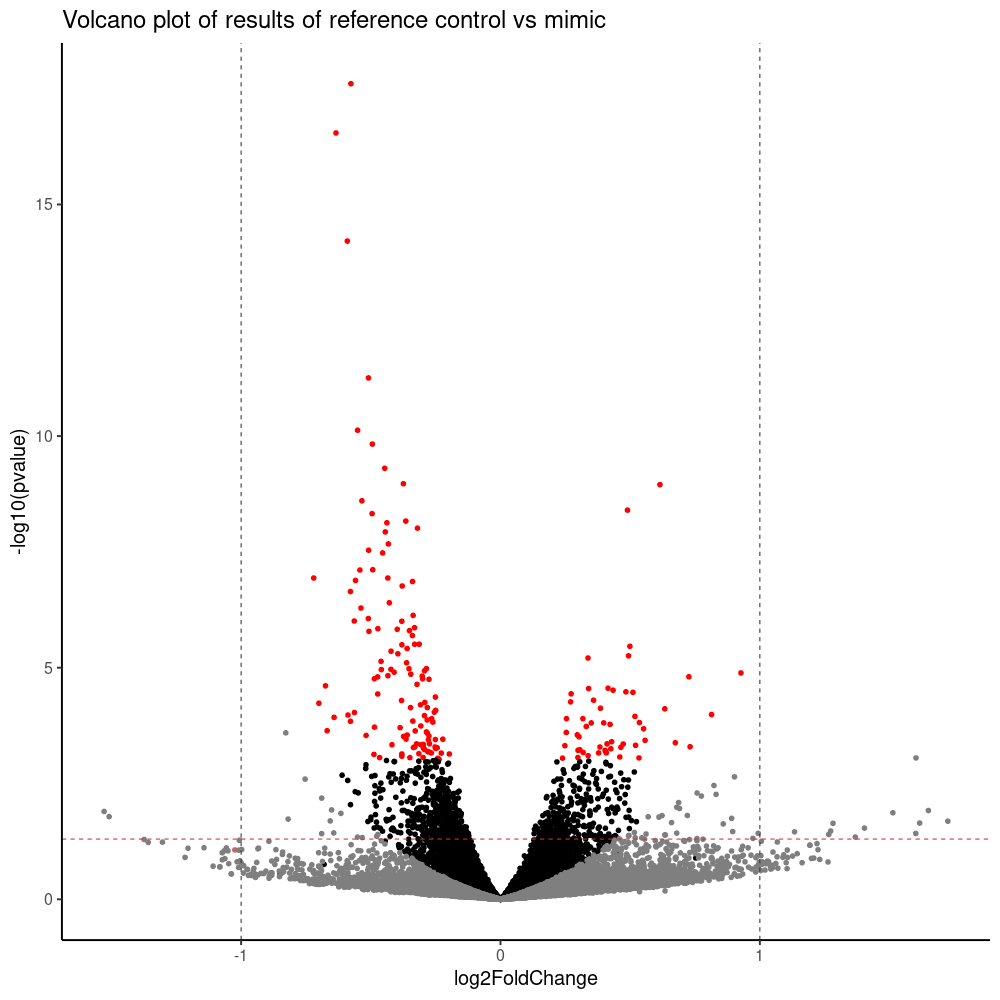
**

**Supplementary table 2**

RNA sequencing results with adjusted p-value <0.05 and minimum fold change of +/- 1.2

| **baseMean** | **log2FoldChange** | **lfcSE** | **stat** | **pvalue** | **padj** | **geneName** |
| --- | --- | --- | --- | --- | --- | --- |
| 6745,490866 | -0,576718308 | 0,066035 | -8,73348 | 2,47E-18 | 2,23E-14 | Ywhag |
| 3190,012477 | -0,634318604 | 0,075047 | -8,45231 | 2,86E-17 | 1,29E-13 | Vat1 |
| 2414,920336 | -0,590538634 | 0,075704 | -7,80058 | 6,16E-15 | 1,85E-11 | Gfpt1 |
| 2950,497328 | -0,508995286 | 0,07387 | -6,89041 | 5,56E-12 | 1,25E-08 | Plau |
| 1581,204041 | -0,550983924 | 0,084625 | -6,51085 | 7,47E-11 | 1,35E-07 | Ehd4 |
| 2695,598349 | -0,494061435 | 0,077119 | -6,40647 | 1,49E-10 | 2,24E-07 | Flt1 |
| 3076,727956 | -0,446345805 | 0,071756 | -6,22035 | 4,96E-10 | 6,39E-07 | Timp3 |
| 9384,7947 | -0,374419798 | 0,061392 | -6,09881 | 1,07E-09 | 1,12E-06 | Ncam1 |
| 1537,777694 | 0,614848543 | 0,100931 | 6,091759 | 1,12E-09 | 1,12E-06 | Trp53inp1 |
| 1311,648394 | -0,534343575 | 0,089631 | -5,96156 | 2,50E-09 | 2,25E-06 | Mcfd2 |
| 3047,295416 | 0,489667168 | 0,083193 | 5,885952 | 3,96E-09 | 3,25E-06 | Ogn |
| 1896,215653 | -0,495108224 | 0,084533 | -5,85697 | 4,71E-09 | 3,54E-06 | Amot |
| 29325,81578 | -0,365074319 | 0,062999 | -5,7949 | 6,84E-09 | 4,74E-06 | Serpine1 |
| 3134,237208 | -0,438009898 | 0,075775 | -5,78044 | 7,45E-09 | 4,80E-06 | Map1b |
| 86766,76796 | -0,320126441 | 0,055814 | -5,73563 | 9,71E-09 | 5,84E-06 | Ahnak |
| 3048,678707 | -0,444090926 | 0,077858 | -5,70384 | 1,17E-08 | 6,60E-06 | Ankrd52 |
| 3336,001027 | -0,432274603 | 0,077171 | -5,60149 | 2,13E-08 | 1,13E-05 | Afap1 |
| 1392,409267 | -0,508369725 | 0,091651 | -5,54682 | 2,91E-08 | 1,46E-05 | Pcyox1 |
| 1708,248435 | -0,454570564 | 0,082306 | -5,52291 | 3,33E-08 | 1,58E-05 | Gnaq |
| 978,2971122 | -0,542077265 | 0,100913 | -5,37174 | 7,80E-08 | 3,35E-05 | Zbtb4 |
| 1474,547918 | -0,49265589 | 0,091667 | -5,37439 | 7,68E-08 | 3,35E-05 | Foxk1 |
| 425,3728089 | -0,720195435 | 0,135888 | -5,29993 | 1,16E-07 | 4,54E-05 | Nipal1 |
| 1946,075196 | -0,434262999 | 0,081937 | -5,29999 | 1,16E-07 | 4,54E-05 | Nek7 |
| 1307,99078 | -0,55889747 | 0,105895 | -5,27783 | 1,31E-07 | 4,91E-05 | Htr1b |
| 14393,71159 | -0,339151797 | 0,06438 | -5,26794 | 1,38E-07 | 4,98E-05 | Cyr61 |
| 3160,181401 | -0,379061918 | 0,072519 | -5,22706 | 1,72E-07 | 5,98E-05 | Tnfaip1 |
| 713,0648731 | -0,578592614 | 0,111785 | -5,17593 | 2,27E-07 | 7,58E-05 | Vti1b |
| 1723,932729 | -0,428563518 | 0,084522 | -5,07042 | 3,97E-07 | 0,000128 | Ppp1r12b |
| 777,6885906 | -0,538134651 | 0,107191 | -5,02034 | 5,16E-07 | 0,00016 | Dynll2 |
| 26475,66673 | -0,336877084 | 0,068061 | -4,94966 | 7,43E-07 | 0,000224 | Flnb |
| 1641,201381 | -0,509616074 | 0,103606 | -4,91879 | 8,71E-07 | 0,000253 | Acsl4 |
| 827,5853178 | -0,563864102 | 0,115189 | -4,8951 | 9,83E-07 | 0,000272 | Shroom3 |
| 2725,78248 | -0,380566484 | 0,077786 | -4,89246 | 9,96E-07 | 0,000272 | Dcbld2 |
| 10741,49178 | -0,331347191 | 0,068612 | -4,82928 | 1,37E-06 | 0,000364 | Tns1 |
| 1063,838689 | -0,472805936 | 0,098102 | -4,81953 | 1,44E-06 | 0,000371 | Lrrc32 |
| 2818,256997 | -0,397981487 | 0,082678 | -4,81361 | 1,48E-06 | 0,000371 | Rbbp7 |
| 2837,975912 | -0,351038687 | 0,07313 | -4,80018 | 1,59E-06 | 0,000386 | B4galt1 |
| 780,8362242 | -0,507010784 | 0,105785 | -4,79285 | 1,64E-06 | 0,00039 | Akt3 |
| 3431,845735 | -0,339636597 | 0,071492 | -4,75067 | 2,03E-06 | 0,000469 | Dpysl2 |
| 4874,098643 | -0,330985324 | 0,070984 | -4,66281 | 3,12E-06 | 0,000686 | Tmsb10 |
| 8087,402178 | -0,313476918 | 0,06722 | -4,66343 | 3,11E-06 | 0,000686 | Actr2 |
| 1904,417899 | -0,380137957 | 0,081616 | -4,65763 | 3,20E-06 | 0,000687 | Spire1 |
| 997,8325881 | 0,498861229 | 0,107468 | 4,641936 | 3,45E-06 | 0,000724 | Vps54 |
| 2242,400586 | -0,36026837 | 0,077991 | -4,61933 | 3,85E-06 | 0,000789 | Myo1d |
| 1264,093413 | -0,421919412 | 0,091899 | -4,5911 | 4,41E-06 | 0,000884 | Snx30 |
| 1595,787936 | -0,395702423 | 0,086692 | -4,56446 | 5,01E-06 | 0,000982 | Tubb2b |
| 765,1299459 | 0,493616379 | 0,108657 | 4,54288 | 5,55E-06 | 0,001065 | Bub1 |
| 3069,641167 | 0,33734669 | 0,074629 | 4,520303 | 6,18E-06 | 0,001161 | Tmtc1 |
| 868,9008068 | -0,460469534 | 0,10269 | -4,48409 | 7,32E-06 | 0,001348 | Gcnt4 |
| 1835,214367 | -0,362088948 | 0,08101 | -4,46968 | 7,83E-06 | 0,001413 | Nras |
| 4791,61322 | -0,352839767 | 0,080083 | -4,4059 | 1,05E-05 | 0,001828 | Prrc2b |
| 7030,30842 | -0,285634848 | 0,064816 | -4,40688 | 1,05E-05 | 0,001828 | Ago2 |
| 918,3337125 | -0,459397982 | 0,10448 | -4,39699 | 1,10E-05 | 0,001834 | Dnajb12 |
| 1162,098678 | -0,422359008 | 0,096009 | -4,39918 | 1,09E-05 | 0,001834 | Map3k3 |
| 6975,307056 | -0,293038008 | 0,066846 | -4,38378 | 1,17E-05 | 0,001913 | Klf6 |
| 1107,438816 | -0,410086832 | 0,09387 | -4,36866 | 1,25E-05 | 0,002014 | Ipo8 |
| 185,6248331 | 0,927016818 | 0,212584 | 4,3607 | 1,30E-05 | 0,002052 | Stc1 |
| 2477,996386 | -0,346035307 | 0,079609 | -4,34671 | 1,38E-05 | 0,002149 | Ptbp3 |
| 952,8432272 | -0,434084773 | 0,100242 | -4,33038 | 1,49E-05 | 0,002276 | Fam171a1 |
| 3643,547223 | -0,301890667 | 0,069797 | -4,32529 | 1,52E-05 | 0,00229 | Lrrfip1 |
| 713,5696993 | -0,473832273 | 0,109756 | -4,31716 | 1,58E-05 | 0,0023 | Trak1 |
| 278,062655 | 0,726369032 | 0,168202 | 4,318425 | 1,57E-05 | 0,0023 | Ahr |
| 687,4894422 | -0,486153825 | 0,113144 | -4,29675 | 1,73E-05 | 0,002453 | Trim2 |
| 6007,712458 | -0,300189034 | 0,069879 | -4,29583 | 1,74E-05 | 0,002453 | Psap |
| 17259,69195 | -0,275617198 | 0,064225 | -4,2914 | 1,78E-05 | 0,002464 | Akap12 |
| 3096,817404 | -0,321994123 | 0,076044 | -4,23432 | 2,29E-05 | 0,003133 | Frmd6 |
| 322,3766375 | -0,674737436 | 0,15996 | -4,21815 | 2,46E-05 | 0,003316 | Zdhhc7 |
| 1110,213445 | 0,414731282 | 0,098946 | 4,191507 | 2,77E-05 | 0,003676 | Gxylt1 |
| 1879,533788 | 0,33982936 | 0,081149 | 4,187699 | 2,82E-05 | 0,003684 | Rrm2 |
| 889,423378 | 0,434084988 | 0,104121 | 4,169053 | 3,06E-05 | 0,003942 | Trib2 |
| 626,6907084 | 0,48346035 | 0,116489 | 4,150275 | 3,32E-05 | 0,004219 | Plagl2 |
| 557,3957875 | 0,510688186 | 0,123229 | 4,144228 | 3,41E-05 | 0,004272 | Skp2 |
| 655,3318948 | -0,473403806 | 0,114735 | -4,12607 | 3,69E-05 | 0,004498 | Emc2 |
| 7069,111437 | 0,272362078 | 0,065989 | 4,127365 | 3,67E-05 | 0,004498 | Cyp1b1 |
| 1482,815499 | 0,358735266 | 0,088491 | 4,053929 | 5,04E-05 | 0,005978 | Atad2 |
| 1395,441083 | -0,381476083 | 0,09418 | -4,05051 | 5,11E-05 | 0,005987 | Carm1 |
| 4910,971415 | 0,270233665 | 0,066979 | 4,034618 | 5,47E-05 | 0,006325 | Top2a |
| 5164,422501 | -0,291759611 | 0,072424 | -4,02847 | 5,61E-05 | 0,006411 | Rgs4 |
| 248,4051159 | -0,700290403 | 0,174246 | -4,01897 | 5,85E-05 | 0,006591 | Bcap29 |
| 2519,334806 | -0,308372037 | 0,077086 | -4,00037 | 6,32E-05 | 0,007043 | Hipk2 |
| 3106,486297 | -0,346791547 | 0,087415 | -3,96718 | 7,27E-05 | 0,007905 | Atp8b2 |
| 5977,671087 | -0,282328078 | 0,071163 | -3,96732 | 7,27E-05 | 0,007905 | Nes |
| 997,5316075 | 0,385527145 | 0,097355 | 3,959998 | 7,50E-05 | 0,008049 | Lmnb1 |
| 348,6577354 | 0,633446412 | 0,160252 | 3,952821 | 7,72E-05 | 0,008197 | Dio2 |
| 404,5331728 | -0,563156964 | 0,144098 | -3,90816 | 9,30E-05 | 0,009534 | Prune |
| 167,034635 | 0,813952037 | 0,209545 | 3,884384 | 0,000103 | 0,010398 | Serpinb6b |
| 364,6852621 | -0,58800987 | 0,151636 | -3,87778 | 0,000105 | 0,010566 | Itga6 |
| 5054,536481 | -0,29325864 | 0,075764 | -3,87067 | 0,000109 | 0,01076 | Loxl3 |
| 467,5435526 | 0,518409553 | 0,134261 | 3,86122 | 0,000113 | 0,011063 | Mmp13 |
| 269,6710161 | -0,6414903 | 0,166631 | -3,84977 | 0,000118 | 0,011468 | 2310067B10Rik |
| 21828,39475 | -0,265463595 | 0,069248 | -3,83351 | 0,000126 | 0,011871 | Itga5 |
| 1845,422003 | 0,317719691 | 0,082859 | 3,834481 | 0,000126 | 0,011871 | Ptx3 |
| 2746,596538 | -0,282640917 | 0,07403 | -3,81795 | 0,000135 | 0,012515 | Zfp36l2 |
| 323,9606685 | -0,578428284 | 0,15211 | -3,80271 | 0,000143 | 0,013042 | Cpne2 |
| 1875,407723 | -0,337897121 | 0,088814 | -3,80453 | 0,000142 | 0,013042 | Tgm2 |
| 1217,749128 | 0,349815402 | 0,092498 | 3,781885 | 0,000156 | 0,013632 | Hist1h1d |
| 813,1157233 | 0,398007138 | 0,105224 | 3,782491 | 0,000155 | 0,013632 | Chfr |
| 382,1986092 | 0,535674466 | 0,141485 | 3,786095 | 0,000153 | 0,013632 | 6030458C11Rik |
| 742,6389801 | 0,422503403 | 0,112274 | 3,76315 | 0,000168 | 0,014554 | Ssh2 |
| 2327,166361 | -0,307115521 | 0,082027 | -3,7441 | 0,000181 | 0,015554 | Tmem123 |
| 3431,436543 | 0,330598261 | 0,088454 | 3,737515 | 0,000186 | 0,015816 | Aspn |
| 471,9894171 | -0,48585098 | 0,130267 | -3,72964 | 0,000192 | 0,016166 | Tmcc3 |
| 1026,76872 | -0,3871128 | 0,103981 | -3,72292 | 0,000197 | 0,016449 | Tubb2a |
| 343,9324908 | 0,551736744 | 0,148775 | 3,708534 | 0,000208 | 0,017253 | Ptprq |
| 227,3177494 | -0,668416113 | 0,181432 | -3,68412 | 0,000229 | 0,018812 | Zfp37 |
| 1496,097457 | -0,328977276 | 0,089349 | -3,68194 | 0,000231 | 0,018812 | Sema4f |
| 2537,596149 | -0,285424639 | 0,077801 | -3,66865 | 0,000244 | 0,019639 | Atp2b1 |
| 2583,321086 | -0,28204 | 0,077222 | -3,65233 | 0,00026 | 0,020564 | Arf6 |
| 2001,211479 | 0,296140115 | 0,081539 | 3,631867 | 0,000281 | 0,022072 | Tmpo |
| 1736,849416 | -0,359802053 | 0,099133 | -3,62948 | 0,000284 | 0,022086 | Tgfbi |
| 410,6354435 | -0,518298469 | 0,143027 | -3,62379 | 0,00029 | 0,022384 | Cdk18 |
| 16293,21433 | -0,275849047 | 0,076275 | -3,61649 | 0,000299 | 0,02283 | Flnc |
| 879,4476604 | -0,373612509 | 0,103445 | -3,61171 | 0,000304 | 0,023059 | Dstyk |
| 1678,988118 | 0,3027834 | 0,083955 | 3,606488 | 0,00031 | 0,023332 | Erlin2 |
| 1259,792379 | -0,363987533 | 0,101763 | -3,5768 | 0,000348 | 0,025847 | Mpp5 |
| 2205,928259 | -0,277898296 | 0,077917 | -3,56661 | 0,000362 | 0,026309 | Edem1 |
| 302,4910187 | 0,557545959 | 0,156628 | 3,55968 | 0,000371 | 0,026796 | Mylip |
| 799,1760804 | 0,428602227 | 0,120999 | 3,542183 | 0,000397 | 0,028411 | Dzip3 |
| 200,3279411 | 0,674120199 | 0,19101 | 3,529242 | 0,000417 | 0,029603 | Kcnj2 |
| 1376,333068 | -0,323553992 | 0,092067 | -3,51431 | 0,000441 | 0,030583 | Zfand3 |
| 2369,025234 | -0,273785565 | 0,077903 | -3,51444 | 0,000441 | 0,030583 | 9430020K01Rik |
| 602,9669525 | 0,410751906 | 0,116872 | 3,514554 | 0,00044 | 0,030583 | Gas2l3 |
| 451,6930647 | 0,473184675 | 0,134719 | 3,51238 | 0,000444 | 0,030583 | Atad5 |
| 1798,067719 | -0,29787007 | 0,084933 | -3,50714 | 0,000453 | 0,030956 | Tsc22d3 |
| 572,3189291 | -0,418337048 | 0,119441 | -3,50245 | 0,000461 | 0,031035 | Met |
| 1415,49523 | -0,306415556 | 0,087477 | -3,50281 | 0,00046 | 0,031035 | Dok1 |
| 348,5317627 | 0,52094657 | 0,149058 | 3,49492 | 0,000474 | 0,031687 | Rasgrp3 |
| 1353,12686 | -0,33070343 | 0,095185 | -3,47431 | 0,000512 | 0,033214 | Diap1 |
| 775,2929735 | 0,383304977 | 0,11038 | 3,472591 | 0,000515 | 0,033214 | Col27a1 |
| 163,2590575 | 0,73100728 | 0,210308 | 3,475889 | 0,000509 | 0,033214 | Ndnf |
| 466,5023396 | 0,46420248 | 0,13382 | 3,468849 | 0,000523 | 0,033441 | Mis18bp1 |
| 1082,928969 | -0,335680575 | 0,096857 | -3,46573 | 0,000529 | 0,033594 | Tnfrsf10b |
| 1697,081159 | -0,297383146 | 0,085909 | -3,46159 | 0,000537 | 0,03364 | Hk1 |
| 1482,161021 | -0,296019425 | 0,085709 | -3,45376 | 0,000553 | 0,034159 | Mfsd1 |
| 528,7703432 | 0,425477672 | 0,123362 | 3,449015 | 0,000563 | 0,034528 | Snap29 |
| 1672,760261 | -0,292318509 | 0,085036 | -3,43758 | 0,000587 | 0,035775 | Scd1 |
| 1305,211727 | 0,306067806 | 0,089104 | 3,434967 | 0,000593 | 0,03588 | Kif11 |
| 1416,324327 | 0,298717278 | 0,087121 | 3,428763 | 0,000606 | 0,036406 | Flrt3 |
| 686,2905303 | 0,403198185 | 0,11764 | 3,427403 | 0,000609 | 0,036406 | Clspn |
| 1809,966416 | -0,279926037 | 0,082071 | -3,41076 | 0,000648 | 0,038011 | Pam |
| 1944,434322 | -0,277893185 | 0,081486 | -3,41031 | 0,000649 | 0,038011 | Maea |
| 662,8032005 | 0,405829031 | 0,118905 | 3,413042 | 0,000642 | 0,038011 | Brwd3 |
| 1120,328499 | 0,318809159 | 0,093812 | 3,398389 | 0,000678 | 0,03945 | Tor1aip1 |
| 32432,93596 | -0,266701504 | 0,078601 | -3,3931 | 0,000691 | 0,039472 | Plec |
| 939,2276559 | 0,378257661 | 0,111482 | 3,392988 | 0,000691 | 0,039472 | Tmx1 |
| 582,1498902 | 0,407974499 | 0,120214 | 3,393732 | 0,000689 | 0,039472 | Gm2115 |
| 1140,536047 | -0,314689974 | 0,093045 | -3,38214 | 0,000719 | 0,040552 | Cdr2 |
| 716,0667636 | -0,380268143 | 0,112531 | -3,37922 | 0,000727 | 0,040568 | Nacc2 |
| 376,1184192 | -0,487954893 | 0,14469 | -3,37243 | 0,000745 | 0,041236 | Reck |
| 934,3981393 | 0,338080119 | 0,100758 | 3,355354 | 0,000793 | 0,0436 | Ckap2 |
| 783,1389192 | -0,380630358 | 0,113689 | -3,34798 | 0,000814 | 0,044504 | Gadd45g |
| 406,2698615 | 0,459863526 | 0,137784 | 3,33758 | 0,000845 | 0,045926 | Tipin |
| 387,4608438 | -0,465971774 | 0,14 | -3,32837 | 0,000874 | 0,046356 | Bend3 |
| 809,5501562 | -0,349416326 | 0,104974 | -3,3286 | 0,000873 | 0,046356 | S1pr1 |
| 2420,193284 | -0,298456791 | 0,089634 | -3,32973 | 0,000869 | 0,046356 | Esyt1 |
| 1404,740556 | 0,298481991 | 0,089638 | 3,329842 | 0,000869 | 0,046356 | Pan3 |
| 300,1753754 | 0,534059497 | 0,160653 | 3,324313 | 0,000886 | 0,04676 | Slfn9 |

**Supplementary table 3**

P values for each figure.

| **Figure 1** | **P value** |
| --- | --- |
| Mmu-mir-181a-5p E11vs E12 | 0.9182 |
| Mmu-mir-181a-5p E11vs E13 | 0.4829 |
| Mmu-mir-181a-5p E11vs E14 | 0.1759 |
| Mmu-mir-181a-5p E11vs E15 | 0.0002 |
| Mmu-mir-181a-5p E11vs E16 | 0.0004 |
| Mmu-mir-181a-5p E11vs E17 | <0.0001 |
| Mmu-mir-181a-5p E11vs E18 | <0.0001 |
| Mmu-mir-181a-5p E12vs E13 | 0.9907 |
| Mmu-mir-181a-5p E12vs E14 | 0.8180 |
| Mmu-mir-181a-5p E12vs E15 | 0.0037 |
| Mmu-mir-181a-5p E12vs E16 | 0.0072 |
| Mmu-mir-181a-5p E12vs E17 | <0.0001 |
| Mmu-mir-181a-5p E12vs E18 | <0.0001 |
| Mmu-mir-181a-5p E13vs E14 | 0.9976 |
| Mmu-mir-181a-5p E13vs E15 | 0.0253 |
| Mmu-mir-181a-5p E13vs E16 | 0.0468 |
| Mmu-mir-181a-5p E13vs E17 | <0.0001 |
| Mmu-mir-181a-5p E13vs E18 | 0.0001 |
| Mmu-mir-181a-5p E14vs E15 | 0.1024 |
| Mmu-mir-181a-5p E14vs E16 | 0.1735 |
| Mmu-mir-181a-5p E14vs E17 | 0.0002 |
| Mmu-mir-181a-5p E14vs E18 | 0.0006 |
| Mmu-mir-181a-5p E15vs E16 | >0.9999 |
| Mmu-mir-181a-5p E15vs E17 | 0.1506 |
| Mmu-mir-181a-5p E15vs E18 | 0.3942 |
| Mmu-mir-181a-5p E16vs E17 | 0.0878 |
| Mmu-mir-181a-5p E16vs E18 | 0.2586 |
| Mmu-mir-181a-5p E17vs E18 | 0.9988 |
| **Figure 2 A** | **P value** |
| Mmu-mir-181a-5p oh vs 24h | 0.1251 |
| Mmu-mir-181a-5p oh vs 48h | 0.0080 |
| Mmu-mir-181a-5p oh vs 7d | <0.0001 |
| Mmu-mir-181a-5p o24h vs 48h | 0.2594 |
| Mmu-mir-181a-5p 24h vs 7d | 0.0001 |
| Mmu-mir-181a-5p 48h vs 7d | 0.0009 |
| Runx2 0h vs 24h | 0.3397 |
| Runx2 0h vs 48h | 0.0047 |
| Runx2 0h vs 7d | 0.0019 |
| Runx2 24h vs 48h | 0.0420 |
| Runx2 24h vs 7d | 0.0147 |
| Runx2 48h vs 7d | 0.8680 |
| Alpl 0h vs 24h | 0.9993 |
| Alpl 0h vs 48h | 0.9999 |
| Alpl 0h vs 7d | 0.0007 |
| Alpl 24h vs 48h | >0.9999 |
| Alpl 24h vs 7d | 0.0008 |
| Alpl 48h vs 7d | 0.0008 |
| **Figure 2 B** | **P value** |
| mmu-miR-181a-5p negative control:24h vs. mimic:24h | <0.0001 |
| mmu-miR-181a-5p negative control:48h vs. mimc:48h | 0.0001 |
| mmu-miR-181a-5p negative control:7d vs mimic:7d | 0.9983 |
| Runx2 negative control:24h vs. mimic:24h | 0.0956 |
| Runx2 negative control:48h vs. mimc:48h | 0.8531 |
| Runx2negative control:7d vs. mimc:7d | 0.9354 |
| Alpl negative control:24h vs. mimic:24h | 0.9377 |
| Alpl negative control:48h vs. mimc:48h | 0.8018 |
| Alpl negative control:7d vs. mimc:7d | 0.9741 |
| **Figure 4** |  |
| Stc1 negative control vs mimic | 0.0156 |
| Col27a negative control vs mimic | 0.4859 |
| Mmp13 negative control vs mimic | 0.0420 |
| Ywhag negative control vs mimic | 0.0270 |
| Amot negative control vs mimic | 0.0134 |
| Mpp5 negative control vs mimic | 0.5490 |
| Frmd6 negative control vs mimic | 0.0380 |
| Serpine1 negative control vs mimic | 0.0281 |
| **Figure 5** |  |
| Stc1 0h vs 24h | 0.7137 |
| Stc1 0h vs 48h | 0.0104 |
| Stc1 0h vs 7d | 0.1599 |
| Stc1 24h vs 48h | 0.0449 |
| Stc1 24h vs 7d | 0.0354 |
| Stc1 48h vs 7d | 0.0007 |
| Mmp13 0h vs 24h | 0.0002 |
| Mmp13 0h vs 48h | 0.0003 |
| Mmp13 0h vs 7d | 0.9443 |
| Mmp13 24h vs 48h | 0.9096 |
| Mmp13 24h vs 7d | 0.0003 |
| Mmp13 48h vs 7d | 0.0006 |
| Frmd6 0h vs 24h | 0.9437 |
| Frmd6 0h vs 48h | 0.0140 |
| Frmd6 0h vs 7d | 0.0637 |
| Frmd6 24h vs 48h | 0.0068 |
| Frmd6 24h vs 7d | 0.0295 |
| Frmd6 48h vs 7d | 0.6984 |
| Amot 0h vs 24h | >0.9999 |
| Amot 0h vs 48h | 0.2018 |
| Amot 0h vs 7d | 0.0708 |
| Amot 24h vs 48h | 0.2168 |
| Ammot 24h vs 7d | 0.0764 |
| Amot 48h vs 7d | 0.8732 |
| Ywhag 0h vs 24h | 0.6470 |
| Ywhag 0h vs 48h | 0.0076 |
| Ywhag 0h vs 7d | 0.0027 |
| Ywhag 24h vs 48h | 0.0018 |
| Ywhag 24h vs 7d | 0.0007 |
| Ywhag 48h vs 7d | 0.8283 |
| Serpine1 0h vs 24h | >0.9999 |
| Serpine1 0h vs 48h | 0.0074 |
| Serpine1 0h vs 7d | 0.0048 |
| Serpine1 24h vs 48h | 0.0074 |
| Serpine1 24h vs 7d | 0.0048 |
| Serpine1 48h vs 7d | 0.9840 |
